# Miniature Uncooled and Unchopped Fiber Optic Infrared Thermometer for Application to Cutting Tool Temperature Measurement

**DOI:** 10.3390/s18103188

**Published:** 2018-09-20

**Authors:** Andrew D. Heeley, Matthew J. Hobbs, Hatim Laalej, Jon R. Willmott

**Affiliations:** 1 Portobello Centre, Sensor Systems Group, Department of Electronic & Electrical Engineering, University of Sheffield, Pitt Street, Sheffield S1 4ET, UK; adheeley1@sheffield.ac.uk (A.D.H.); m.hobbs@sheffield.ac.uk (M.J.H.); 2 Advanced Manufacturing Research Centre (AMRC) with Boeing, Machining Research, Process Modelling and Control Group, Factory of the Future, Wallis Way, Advanced Manufacturing Park, Catcliffe, Rotherham, South Yorkshire S60 5TZ, UK; h.laalej@sheffield.ac.uk

**Keywords:** infrared thermometer, mid-wave infrared, indium arsenide antimony photodiode, uncooled thermometer, fiber optic coupling, machining temperature measurements

## Abstract

A new infrared thermometer, sensitive to wavelengths between 3 μm and 3.5 μm, has been developed. It is based on an Indium Arsenide Antimony (InAsSb) photodiode, a transimpedance amplifier, and a sapphire fiber optic cable. The thermometer used an uncooled photodiode sensor and received infrared radiation that did not undergo any form of optical chopping, thereby, minimizing the physical size of the device and affording its attachment to a milling machine tool holder. The thermometer is intended for applications requiring that the electronics are located remotely from high-temperature conditions incurred during machining but also affording the potential for use in other harsh conditions. Other example applications include: processes involving chemical reactions and abrasion or fluids that would otherwise present problems for invasive contact sensors to achieve reliable and accurate measurements. The prototype thermometer was capable of measuring temperatures between 200 °C and 1000 °C with sapphire fiber optic cable coupling to high temperature conditions. Future versions of the device will afford temperature measurements on a milling machine cutting tool and could substitute for the standard method of embedding thermocouple wires into the cutting tool inserts. Similarly, other objects within harsh conditions could be measured using these techniques and accelerate developments of the thermometer to suit particular applications.

## 1. Introduction

Temperature measurement is fundamental to understanding the performance of many manufacturing processes and products. Materials machining is one of the most commonly used manufacturing techniques [1], and the efficiency of the process and quality of the final product are both affected by the temperatures achieved. A thorough understanding of the relationships between temperature, process parameters, and product relies upon acquiring accurate, or at least, representative temperature measurements during manufacture [1,2,3,4]. The need to improve machining process efficiency and product quality affords motivation for research into improved methods of temperature measurement during machining.

Thermocouples have been the standard thermometer used for many industrial measurements, including temperature measurement during material machining. The principal drivers for using thermocouples have been the low capital and installation costs incurred and the wide range of temperatures that can be measured and the ease of deployment. The implementation of thermocouple-based temperature measurement has weaknesses, such as: requiring invasive contact, whilst also having low sensitivity; low chemical resistance; very high rates of calibration drift, particularly at high temperatures; and accuracy limitations [4]. These limitations have been ameliorated for many installations, allowing the acquisition of reliable measurements [5].

Infrared radiation thermometers overcome many of the limitations of thermocouples and particularly those arising from having invasive contact between the sensor and the object being measured [6,7], thereby obviating the problems related to having the sensor in harsh conditions.

One field of application that requires precise temperature measurements, that are acquired quickly under harsh conditions, is machine tool surface temperature measurement [8,9,10,11]. Machining tool surface temperature measurements have often been acquired using embedded thermocouples [12,13,14,15] and increasingly by infrared thermography and thermometry [8,9,16,17,18,19,20,21,22]. Infrared thermometers have been used for measuring temperatures with the sensor embedded into the tool or positioned adjacent to the surface [8,9,14]. Other researchers have attempted to measure machining process temperatures using externally mounted infrared thermometers viewing the machined surface [16,21,23]. Machining imposes physically harsh conditions on the tools and any measurement equipment used owing to the high and cyclic temperatures, abrasive materials, cutting oil contaminants, impact from the cutting tools striking the work-piece, and the potential risk of damage from the impact of ‘swarf’ (fragments of metal that are cut from a work-piece).

Infrared thermometers have been tested under laboratory conditions and afforded good temperature measurements in these circumstances; however, fewer systems have been utilized within the manufacturing processes [2], owing to the difficulties of expediting the sensors in such applications [5,14,24]. With increased research efforts dedicated to measuring temperatures accurately, more robust systems have been developed. These have tended to be used where the cost of machine failure exceeded the cost of acquiring the measurement, for example, measuring in-service gas turbine blade temperatures [7].

Typical infrared thermometers use a photosensitive sensor, for example, a semiconductor photodiode with an electronic circuit to interpret the sensor response to incoming radiation as a temperature measurement [6]. A number of infrared thermometers also use fiber optic coupling of the infrared radiation from the measured object to the sensor; enabling positioning of the sensor remote from the source [8,10,25]. The usage of suitable fiber optic cables increases the capital and installation costs of a thermometer, therefore, a more limited range of applications exist for which these more specialized optics can be justified readily [6,26].

A suitable thermometer for measuring the temperature of a cutting tool surface would use an infrared transmitting fiber optic cable embedded into the rear of the cutting tool ‘insert’ (the part of the tool that performs the cutting action), and may have the signal processing electronics positioned remotely. The range of temperatures incurred within cutting processes dictates that the thermometer should respond to mid-wavelength infrared radiation (MWIR, typically defined as 3–8 μm), and that the short duration over which the tool would be cutting dictates that the thermometer must have a high speed of response.

The ‘blind’ hole into which the fiber optic would be inserted would have a length to diameter ratio exceeding 5, and the fiber optic core would be exposed along the embedded length, thereby providing a means of collecting radiation from the full surface area of the hole. The depth to bore ratio of this blind hole and the exposed fiber optic would obviate the problem of having to compensate the measured signal for the effect of the relative emissivity of the radiating object because the conditions approximate a blackbody, achieving very high relative emissivity approaching unity [27,28].

Sapphire fiber optics are capable of operating at the highest temperatures likely to be incurred during cutting and provide very good resistance to attack and degradation by chemical species [29]. The cables are also brittle; therefore, require protection from excessive impact, tension, or torsion forces.

Infrared thermometers intended for accurate temperature measurements at low signal intensity use techniques to improve the accuracy and stability of the measurements acquired [6,30], typically: optical chopping of the incoming radiation to provide intermittent dark current measurement, thereby, affording offset compensation; thermoelectric cooling of the sensor to minimize the detector dark current and its drift, and increase the photodiode shunt resistance.

Accommodating these features necessitates an increased thermometer enclosure size and additional electronics for controlling the cooling and undertaking additional signal processing [30]. Development of an infrared thermometer that used an uncooled sensor and received the radiant signal without optical chopping, but had the capability of measuring machine tool temperatures, would advance machining tool temperature measurement [8,11].

This work introduces a thermometer that has been based upon using an infrared fiber optic cable to transfer radiation from an object, positioned within a region of harsh conditions, to a semiconductor photodiode. Uniquely, this has been achieved without optical chopping or cooling of the photodiode; thereby, enabling the practical application of radiation thermometry to this application. The thermometer was tested over the temperature measurement range 100 °C to 1000 °C under laboratory conditions. This range of temperatures is useful for a thermometer intended for measuring material cutting temperatures [2,15].

## 2. Materials and Methods

### 2.1. Description of the Thermometer

An uncooled Indium Arsenide Antimony (InAsSb) photodiode of type Hamamatsu 13243-11-MA (Hamamatsu Photonics, Hamamatsu, Japan) [31] was chosen because the signal processing circuit and thermometer instrument could be made in a compact size. It was concluded from the detector specifications that it may be possible to construct an instrument without the need for bulky optical chopping or cooling of the detector. The photosensitivity of this photodiode was lower than other candidate photodiodes; however, its availability in a compact transistor outline (TO) type, TO-46 package favored its usage in our example application.

The thermometer comprised a sapphire fiber optic cable that coupled infrared radiation from a source, to the photodiode. The photodiode operated over a nominal wavelength range of 3.0 to 5.0 μm and had an active area of 0.7 mm × 0.7 mm. A transimpedance amplifier (TIA) was used to convert the photocurrent to a proportional output voltage that could be measured using standard laboratory instrumentation. The thermometer used a 1 m length of 425 μm diameter crystalline sapphire fiber optic cable, with a nominal transmission band of 0.75 to 3.5 μm, defined as the band between which the signal attenuation is less than 3 dB/km [29]. The fiber optic coupled infrared radiation from a temperature source, directly to the uncooled photodiode, via an SMA905 [32] that was mounted on the fiber optic cable.

The expected temperature range of the machine tool surface was nominally 100 °C when rotating in free air, to approximately 900 °C when cutting [1]. The temperature expected during cutting necessitated using sapphire fiber optic cable as the embedded sensor, since other fiber optic types would fail at elevated temperatures [33]. Combining the spectral characteristics of the detector and optical fiber, the instrument was sensitive to wavelengths between 3 μm and 3.5 μm. Although the combination of fiber optic and photodiode defined a narrow-band optimum transmission window, transmission continued beyond the nominal roll-off sensitivity of the photodiode and transmission range of the fiber optic, albeit with increased attenuation and lower responsivity.

The photodiode was operated in photovoltaic mode with a boot-strapping circuit intended to elevate the input impedance presented to the signal amplifier. The photocurrent from the photodiode was passed into a conventional TIA circuit with a 1 MΩ transimpedance feedback resistor, to measure a proportional output voltage. The output voltage of the TIA was measurable using a bench-top digital multimeter (DMM) (Keysight Technologies Malaysia, Penang, Malaysia). The inverting input of the TIA, to which the photodiode was connected, employed the boot-strapping technique, demonstrated for measuring the photocurrent from MWIR photodiodes by Makai and Makai [34,35,36] and extended to other photodiodes by Eppeldauer and Martin [37]

The TIA stage with a resistor-capacitor (RC) feedback network had a 1 MΩ transimpedance feedback resistor and a time constant of *τ* = 10 μs; therefore, a cut-off frequency of *f_c_* = 15.9 kHz. The maximum frequency of the signal expected from the milling machine measurements was 1 kHz; therefore, the circuit was capable of acquiring and processing the signal without limiting the measurement. The printed circuit board (PCB) footprint was miniaturized (25 mm × 25 mm footprint) to fit within space constraints of the application.

The manufacturer’s specification for photodiode shunt resistance was of order 120 kΩ at −10 mV bias applied across the photodiode junction and 25 °C ambient temperature. The diode shunt resistance was specified to reduce at a mean rate of 10 kΩ/°C as the diode temperature increased, for example, if it was exposed to an external heat source or became heated by current flow [31].

**Figure 1:**
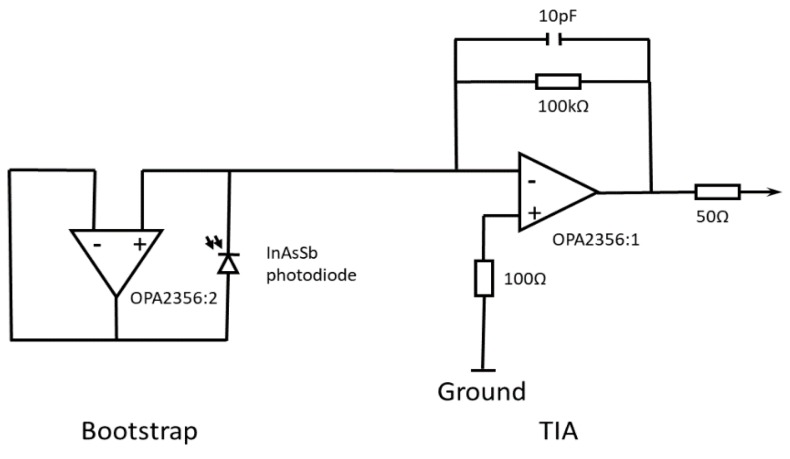
InAsSb photodiode bootstrapped transimpedance amplifier schematic.

The circuit schematic diagram is presented in Figure 1.

**Figure 2:**
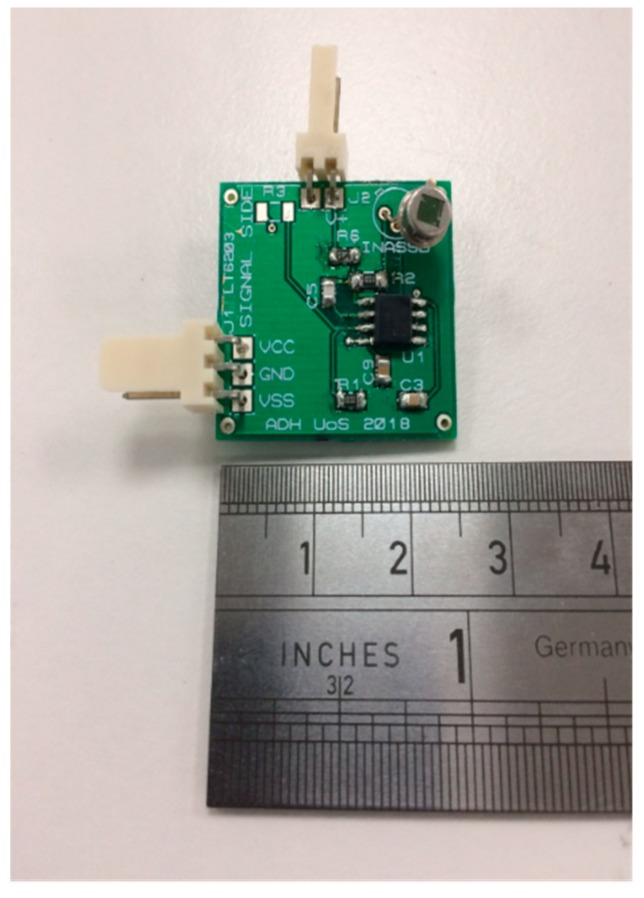
Illustration of PCB size for uncooled InAsSb photodiodes.

The TIA circuit size and detector package size for the uncooled InAsSb photodiode is presented in Figure 2, which illustrates the compactness achieved for the thermometer, by eliminating cooling and chopping. Typical component sizes to implement photodiode cooling and optical chopping: cylindrical heat sinks of order 20 mm diameter; cooling control components of order 50 mm × 50 mm; and optical chopping mechanisms of order 50 mm × 25 mm. Integrated thermoelectric cooling of the InAsSb photodiode would increase the package to a TO-8 size, thereby approximately tripling the height and diameter of the package compared to the TO-46 package of the uncooled photodiode. Including such components could not be achieved without increasing the size of the photodiode package and thermometer enclosure significantly.

The thermometer was constrained to measuring a maximum source temperature of 1000 °C for the application targeted and to ensure compatibility with a commercial inductively-coupled data transmission and external amplifier system that was used during trials of the thermometer mounted inside a milling machine [38].

### 2.2. Calculation of Thermometer Response Expected

**Figure 3:**
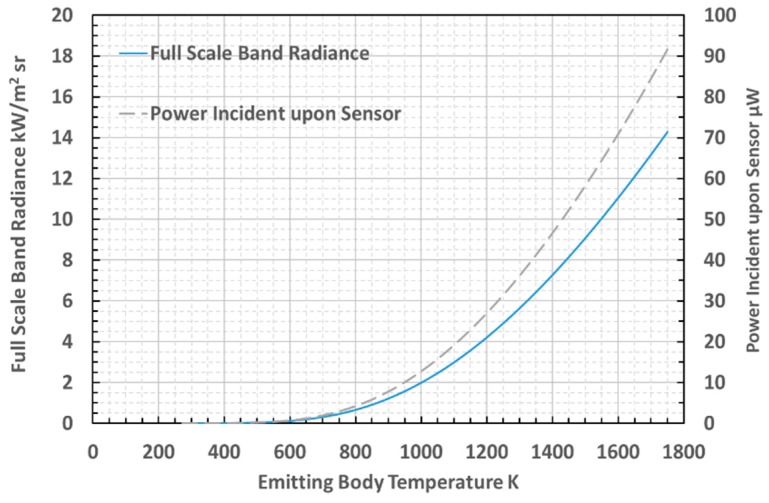
Calculated full scale band radiance and power incident upon InAsSb photodiode.

Prior to calibrating and testing the thermometer, the power of the incident radiation upon the sensor was estimated using numerical integration of Planck’s Law, to evaluate the band radiance over the range of wavelengths 3.0–3.5 μm, at which the maximum power transfer would be expected [39]. The calculated curves of full scale band radiance and power incident upon the photodiode within the 3.0–3.5 μm waveband are presented in Figure 3.

The minimum photocurrent measurable from the InAsSb photodiode was specified, by the manufacturer, to be 150 pA at 0.15 μW radiance incident upon the 0.49 mm^2^ photosensitive area of the sensor [31]. Calculations showed that this photocurrent would generate a transimpedance voltage of 0.15 mV arising from the TIA circuit used in the thermometer. The minimum radiant intensity measurable by this sensor has been calculated to be 0.30 W/m^2^. The minimum photocurrent and band radiance can be interpreted to yield an equivalent minimum temperature that can be measured of 550 K (277 °C). This minimum temperature would enable the measurement of the tool surface temperature cycles during milling operations; which would provide us with much of the thermal information required for our application [2].

### 2.3. Thermometer Blackbody Characterisation and Laboratory Testing

An approximate blackbody furnace (AMETEK Land P1200B, AMETEK Land Instruments, Dronfield, UK) was used to characterize and assess the basic performance parameters, electronic noise as a function of temperature, and working temperature measurement range, of our newly constructed thermometer. The source and ambient temperatures were recorded using an ISOTECH milliK precision thermometer (Isothermal Technology, Southport, UK) with transfer standard type R thermocouple and transfer standard Platinum Resistance Thermometer (PRT), having a resistance of 100 Ω at 0 °C (PRT-100). The milliK thermometer, thermocouple, and PRT-100 were all calibrated to traceable standards by the manufacturer. The fiber optic cable was mounted in a length of lens tube to protect it from external influences and had its open-ended tip inserted into a thermo-well on the blackbody source, to collect infrared radiation. A transfer standard platinum resistance thermometer and thermocouple measured the respective ambient and blackbody source temperatures during characterization of the infrared thermometer. The blackbody source temperature monitoring thermo-well, used for the infrared thermometer, was concentric with the calibration thermo-well, but positioned on the outer diameter of the refractory tube, rather than collinear with the internal target. The depth to diameter ratio of the temperature monitoring thermo-well exceeded 5:1 and the fiber end was exposed to the radiant chamber along a length, such that the length to diameter ratio exceeded 5:1. This configuration afforded a very high relative emissivity approaching ideal blackbody conditions. The characterization and application both had sufficiently large depth to diameter ratios of the blind holes into which the fiber optic was inserted and gathered radiation from a length of fiber to afford validity to the assumption of unit emissivity. The blackbody source reached equilibrium at temperature set point before measurements were acquired for thermometer characterization, thereby, source temperature uniformity was ensured and the transfer standard insertion probe measurement was assumed to be representative of the temperature to which the thermometer was exposed.

The thermometer response to blackbody source temperatures between 100 °C and 1000 °C was recorded using Keysight 34465A data-logging digital multimeters (Keysight Technologies Malaysia, Penang, Malaysia), with the temperature incremented in 50 °C steps. The initial sets of source temperature versus thermometer output data were recorded as the instantaneous values at an interval of 0.5 s between sequential data. The mean and standard deviation of the output voltage from the thermometer were evaluated, to determine the temperature resolution and noise of our thermometer, for each temperature set point. Measurements of the TIA voltage output with the thermometer withdrawn from the blackbody source thermo-well and the sensor covered with an ambient temperature mask were undertaken at the start and end of each set of measurements recorded to achieve a ‘zero’ offset. The mean zero offset for each test was subtracted from every measurement point and the resultant voltage was used as the characteristic output for the source temperature measured.

Measurements were undertaken subsequently, to evaluate the characteristics of the output of the thermometer, with various orifice sizes positioned between the source and thermometer, to assess the field of view (FOV) and proportion of the photodiode area upon which transmitted radiation was incident. The fiber optic was withdrawn from thermo-well and positioned collinearly with and centered upon the blackbody aperture center, thereby affording view of the blackbody source target. The FOV was defined as the aperture diameter at a known position (in this case 68 mm from the fiber optic tip to the target aperture) for which 90% of the maximum signal intensity was recorded.

**Figure 4:**
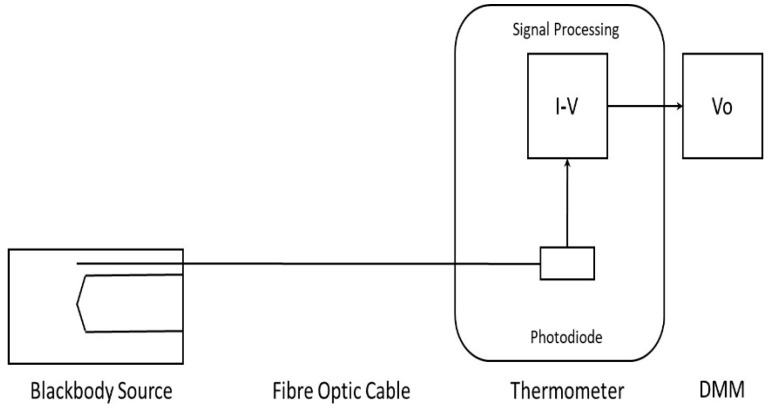
Configuration of laboratory testing of fiber optic thermometer.

The configuration of the laboratory-based testing is presented in Figure 4.

This working calibration or characterization of the prototype infrared thermometer also tested the capability of the sapphire fiber optic cable, photodiode, and TIA circuit combination to measure infrared radiation through the temperature range that was expected to be incurred during measurements within a milling machine.

## 3. Results and Discussion

### 3.1. Thermometer Temperature Measurement versus Output Voltage Characteristics

**Figure 5:**
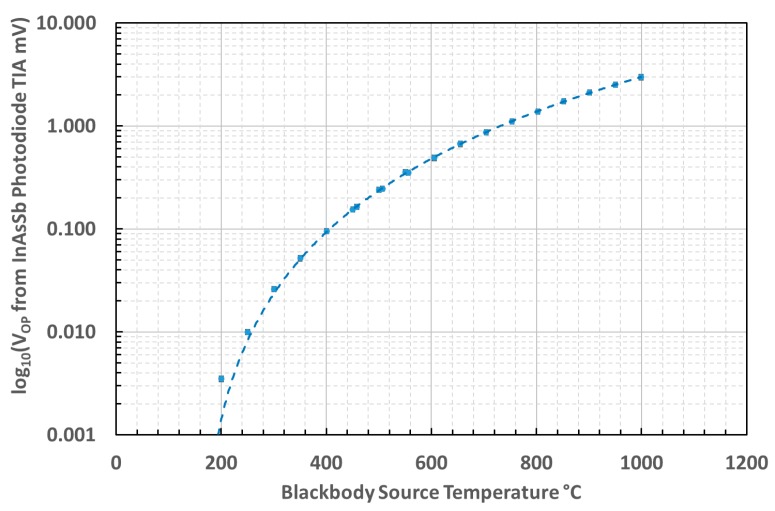
Output voltage of the infrared thermometer as a function of source temperature.

The output voltage characteristic of the thermometer, as a function of source temperature, between 100 °C and 1000 °C is presented as a logarithmic-linear scaled graph in Figure 5.

**Figure 6:**
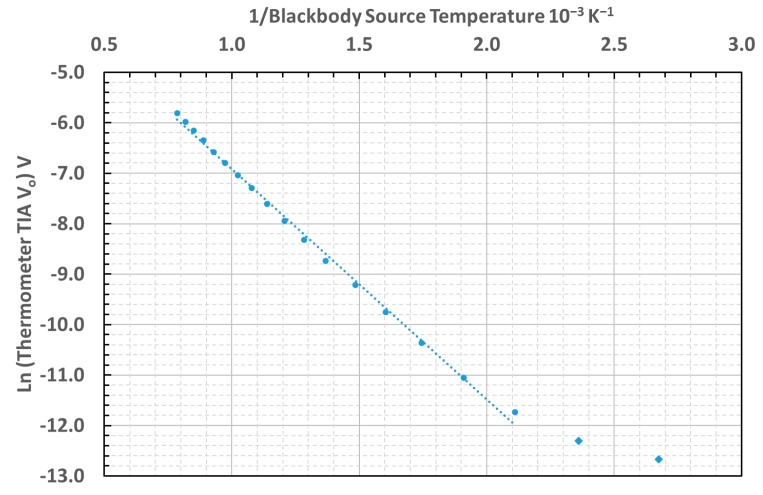
Characteristic inverse absolute temperature of blackbody versus Ln (TIA output voltage) curve for the thermometer.

The linearity of the thermometer was assessed from the characteristic curve of inverse source temperature versus natural logarithm of the thermometer TIA output voltage that should be expected from differentiating the Wien approximation of Planck’s law [40,41]. The characteristic curve measured for this thermometer is presented in Figure 6.

The lowest temperatures tested, between 100 °C and 200°C, deviated from the linear relationship on the graph, owing to the prevalence of noise and offset over the signal at these temperatures.

Our measurements of the unchopped signal from an uncooled InAsSb photodiode demonstrated that the minimum temperature measurable before the noise and offset exceeded the useful signal was circa 200 °C.

The mean effective wavelength of the thermometer was estimated to be 3.15 μm. This was based upon the gradient of the inverse source temperature versus Ln (Thermometer TIA V_o_) characteristic curve, plotted between the inverse of the absolute temperature of the blackbody, and the natural logarithm of the output voltage, above the minimum measurable temperature [40,41].

### 3.2. Evaluation of Steady-State Noise Limited Resolution, Signal to Noise Ratio and Minimum Resolvable Temperature

**Figure 7:**
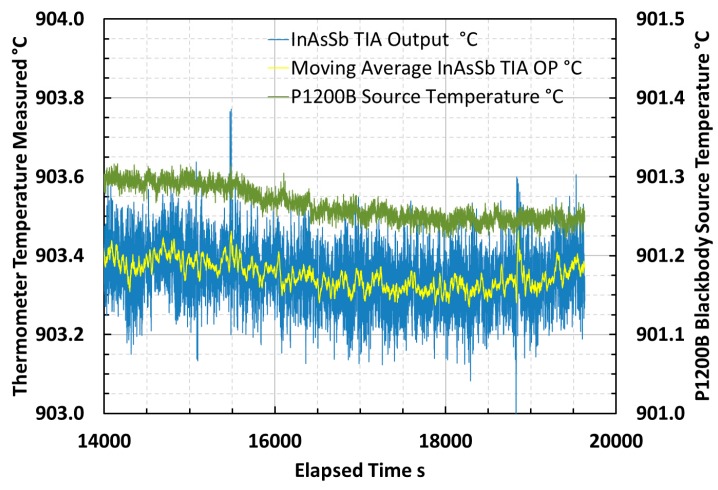
Noise in sampled thermometer signal and continuously-averaged mean thermometer signal, measuring steady-state source temperature of 900 °C.

An example portion of the sampled signal, the continuously-averaged signal over twenty contiguous readings and the temperature variation of the blackbody source at a steady-state temperature of 900 °C are presented in Figure 7. This example is typical of the measurements across the temperature range that was analyzed for mean and standard deviation around the mean, to characterize the output voltage signal and noise of the thermometer during laboratory tests.

**Figure 8:**
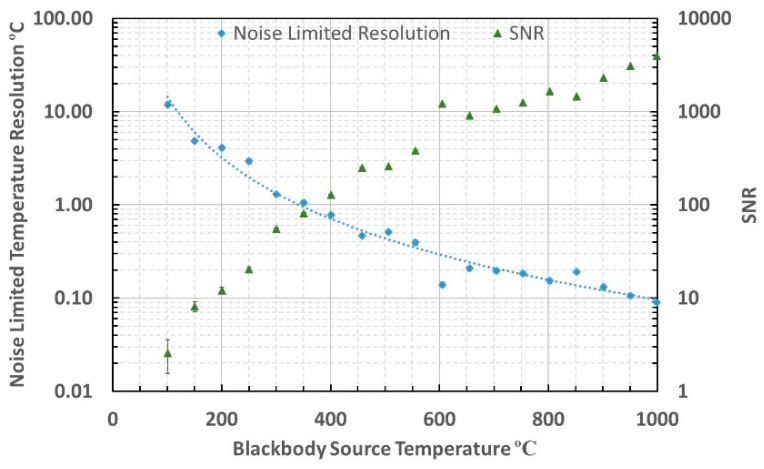
Noise Limited resolution and SNR at blackbody temperatures between 100 and 1000 °C.

The thermometer noise limited resolution and signal to noise ratio (SNR) were evaluated at blackbody source temperatures between 100 °C and 1000 °C using a method based upon the sample mean and standard deviation of the signal. The SNR was defined as the ratio of the sample mean and the sample standard deviation of the signal during each measurement. The noise limited resolution was defined as the Celsius temperature equivalent to the sample standard deviation of the signal, approximated at the mean effective wavelength [40,42,43]. Example raw data is shown in Figure 7. The thermometer noise limited resolution and SNR were evaluated and are presented in Figure 8.

The root mean square (RMS) noise in the thermometer measurements across the arbitrary range of source temperatures 150 °C to 1000 °C was evaluated to be 0.82 mV and the equivalent temperature error was calculated to be 4.4 °C across this range of measurement. The RMS noise limited resolution was 0.8 °C between the minimum temperature for which the measurement achieved SNR of unity, 200 °C, and the arbitrary maximum temperature of 1000 °C.

The minimum temperature that could be measured using this thermometer, based upon achieving unity SNR, was estimated to be approximately 200 °C. The slightly lower than calculated minimum temperature measurable arose because there would have been additional power incident upon the photodiode from outside of the nominal 3.0–3.5 μm waveband upon which the calculation was based.

### 3.3. Field of View Measurement

**Figure 9:**
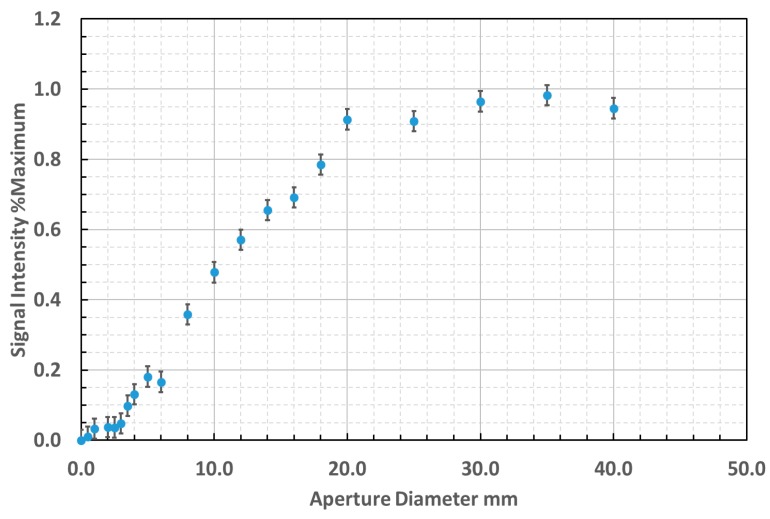
Field of view measurement with aperture positioned at 68 mm from fiber optic cable.

The field of view measurements, normalized with respect to signal intensity, are presented in Figure 9 and demonstrated an aperture of approximately 20 mm at 68 mm target distance. This yielded a FOV of 3.4:1 to 90% power and an equivalent planar included angle of the acceptance cone of 20.8°. The conical angle measured contrasts with the specification for the numerical aperture of the fiber optic of 0.26, equivalent to 25.4° planar included angle of the acceptance cone. It may be inferred that the active area of the photodiode was overfilled by 48% in this configuration by comparison of these geometries.

### 3.4. Comparison of Calculated and Measured Thermometer Output

**Figure 10:**
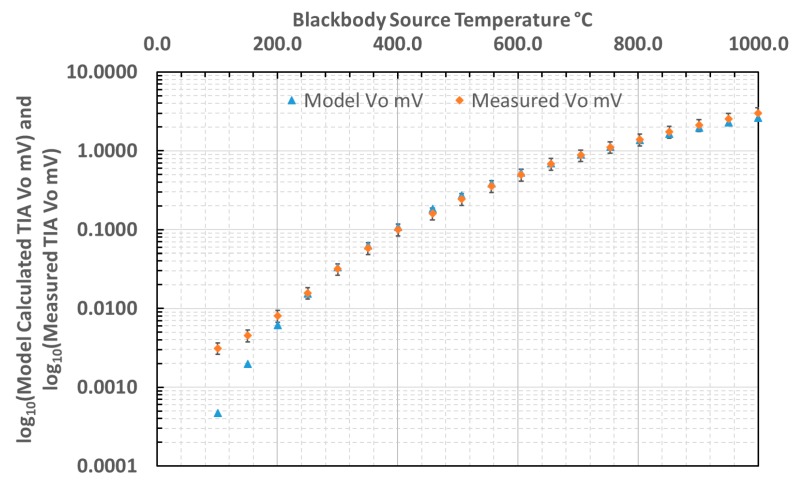
Comparison of calculated and measured output from our thermometer.

The output voltage characteristic of the thermometer, measuring a blackbody source, was both measured and predicted. The predicted curve was based upon a convolution of radiance—itself evaluated by integrating Planck’s Law over the relevant bandwidth—with the detector spectral response. The validity of the thermometer measurement has been assessed by evaluating the error between the calculated and measured voltage output, as presented in Figure 10.

**Figure 11:**
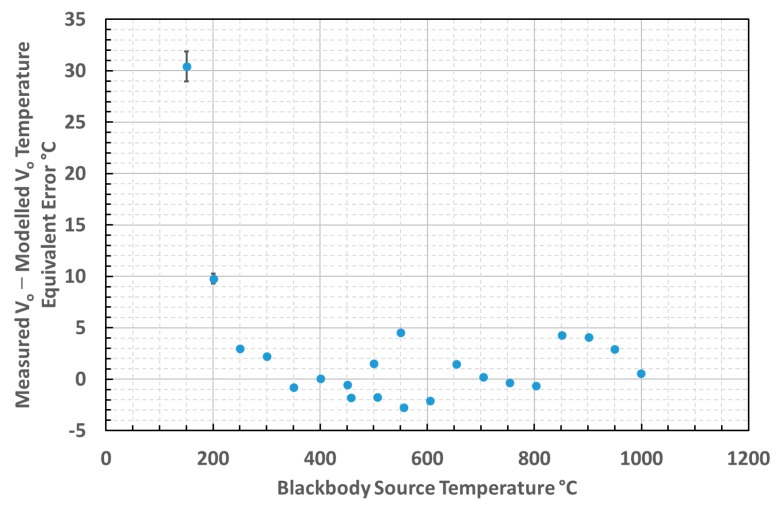
Temperature equivalent error between calculated and measured thermometer output.

The difference between the calculated and measured output voltages from the thermometer across the blackbody temperature range measured was evaluated and translated into an equivalent temperature error. The equivalent temperature errors across the temperature range 150–1000 °C are presented in Figure 11.

### 3.5. Measurement Uncertainty Estimate

The uncertainty budget for single measurements acquired using this thermometer system has been estimated, based upon the individual contributions from the equipment used in calibration and measurement, and from the measurements acquired using the thermometer. The estimated contributions considered in the estimate and arising from the equipment and from the measurement are presented in Table 1 and Table 2, respectively.

The overall uncertainty varied from 2.4% at 200 °C to 2.3% at 1000 °C. The largest individual contributions to the uncertainty arose from the variability in the infrared thermometer measurements and the error arising from the interpolation used to convert between the voltage and temperature in usage. The uncertainties that arose from the calibration equipment can be neglected without detriment to the uncertainty estimate, therefore we can simplify the uncertainty estimation of the thermometer by evaluating only the uncertainty arising from the in-use variability of the measurements and interpolation error, and reporting this sum as the uncertainty of the thermometer.

### 3.6. Example Application: Initial Trial of Temperature Measurements from End Milling Machine

**Figure 12:**
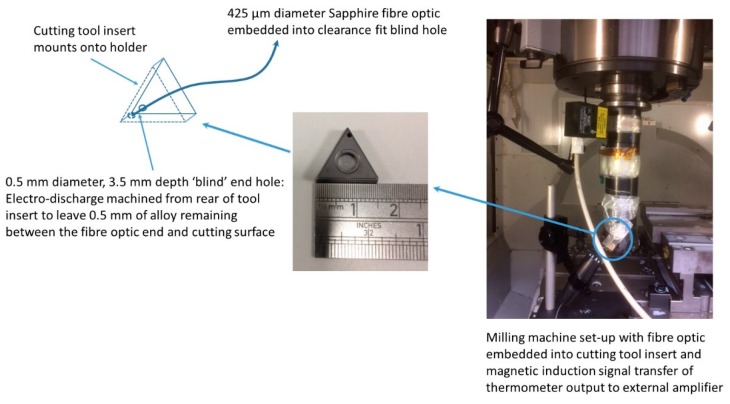
Milling machine set-up and schematic illustration of cutting tool insert configuration.

A version of the thermometer was mounted onto an end milling machine tool holder during cutting trials as an example application of this thermometer. The open end of a fiber optic was embedded into a cutting tool insert and led out to the thermometer. A photograph of the milling machine set-up used during the trial and a schematic diagram of the cutting tool insert configuration with the fiber optic embedded into the blind hole are presented in Figure 12.

**Figure 13:**
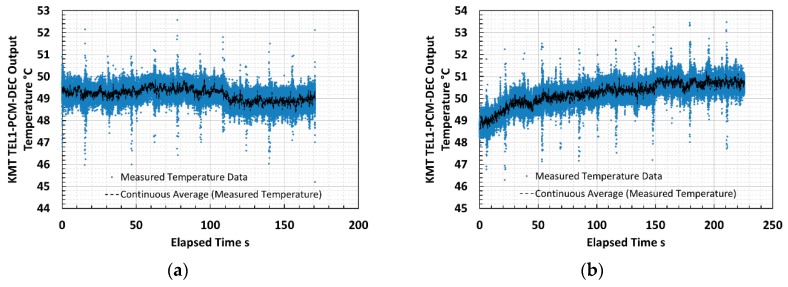
Cutting tool insert temperature recorded by fiber optic thermometer on (**a**) first cutting pass at minimum temperature measurable and (**b**) second cutting pass illustrating marginal temperature increase during milling trials.

Thermometer output measurements were logged via the inductive data transmission system and recorded using LabVIEW, configured as a data logger. The thermometer measured for two consecutive passes of the milling head along a titanium trial plate whilst cutting a simple planar step from the surface. The cutting tool temperatures recorded by the thermometer, along with the continuous mean over 100 data points, during the first and second cutting passes of trial milling, are presented in Figure 13a,b. The continuous mean values were evaluated because the thermometer measurements were noisy (SNR near to unity). The temperature during these early passes was low and at the bottom end of the working range of the thermometer.

The output voltage signals from the thermometer incurred low SNR at the low temperatures experienced during early passes of the milling head; therefore, the traces illustrated were subject to noise, and continuous mean values were evaluated to identify genuine signal variations. The continuously averaged mean of the thermometer output during the second pass demonstrated an increasing signal towards a plateau as the cutting tool progressed along the plate. This form was interpreted as the temperature of the tool insert increasing during the pass. The continuously averaged mean signal, after external amplification at voltage gain G = 250, increased by approximately 20 mV during the pass. A cutting tool temperature increase of approximately 2 °C may be inferred by applying the characteristic approximation derived for the thermometer during laboratory testing.

**Figure 14:**
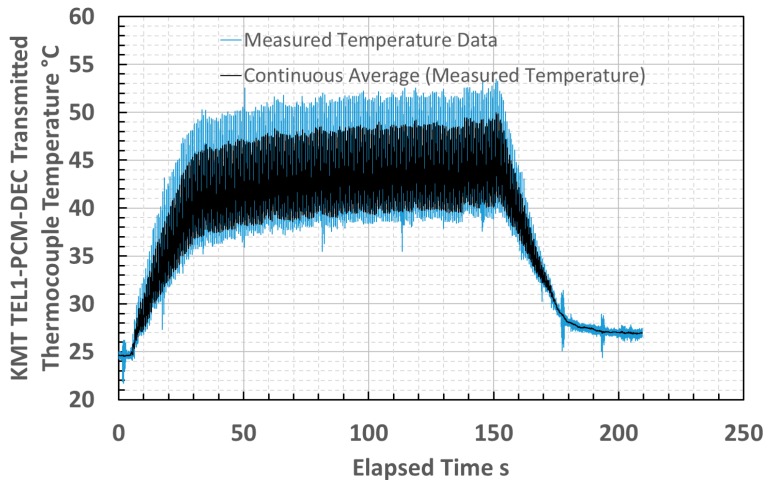
Cutting tool insert temperature measured by an embedded thermocouple from an early pass during end milling trials, illustrating broad comparability with temperatures measured by the prototype infrared thermometer.

Temperature measurements acquired from a different cutting tool insert, cutting from a different volume of the same titanium plate used for the thermometer trial and measured by the standard practice of a type K thermocouple embedded into the insert are presented in Figure 14.

The similarity of the temperatures that were measured by the prototype thermometer and thermocouple and the shape of the curves tending to plateau indicated that the infrared thermometer measurements were realistic. The thermometer did not measure the full duration of the cutting pass because the fiber optic was sheared by stray debris during the pass. This comparison suggested that using a more robust thermometer could afford milling machine tool temperature measurements. The thermocouple and thermometer measurements were not acquired simultaneously, owing to limitations upon transmitting multiple signals from the milling machine. Whilst the machining configurations used were the same to ameliorate the effects of uncontrolled variables between tests, it is only possible to infer the general comparability suggested between the sets of measurements.

## 4. Conclusions

We have demonstrated what we believe to be the first photovoltaic MWIR fiber optic thermometer that has no cooling of the infrared detector or optical chopping. It afforded measurements across the temperature range 200–1000 °C, which is suitable for our example application of machine tool surface temperature measurements. The footprint of the thermometer developed was sufficiently small to afford its use for measurements in applications where installation space would be constrained or a heavy mass would prove impractical, such as attached to the machine spindle.

A minimum temperature of 200 °C was measurable, with peak-to-peak noise magnitude of order 20 μV (order 10 mV after external amplification for data logging) representing unity SNR, below which the signal could not be isolated from the noise.

The RMS noise limited resolution of the thermometer was estimated to be 4.4 °C over the range of temperatures for which the thermometer was characterized, that is, between 150 °C and 1000 °C. The thermometer achieved lower RMS noise when used to measure at higher temperatures, achieving 0.8 °C across the temperature range from 200 °C to 1000 °C. This lower RMS noise suggested that the infrared thermometer could be considered most applicable to substitute for thermocouples at the higher temperatures within its working range.

The noise magnitude and minimum temperature resolvable by this thermometer configuration would need to be assessed for shorter sample durations to understand the scope to compromise between achieving sufficiently fast speed of response and lower noise limited resolution.

The capability of lower noise components, along with different wavelength ranges available from alternative photodiodes and components will be investigated in further work to develop the thermometer. These developments will be aimed at satisfying the constraints and requirements of other applications and temperature ranges, to provide fiber optic coupled thermometers that could afford remote measurement capabilities for difficult conditions, whilst presenting a small physical size.

## Figures and Tables

**Table 1:** Contributions of calibration uncertainties to overall measurement uncertainty.

Source Temperature °C	Calibration Thermocouple Uncertainty%	Calibration Blackbody Uncertainty%	Calibration Instrument Uncertainty%	Digital Multimeter Uncertainty%
200	0.4	0.14	0.2	0.0085
1000	0.08	0.03	0.04	0.0085

**Table 2:** Contributions of measurement uncertainties to overall measurement uncertainty.

Source Temperature °C	Infrared Thermometer Variability in Usage%	Interpolation Error of Thermometer Mean Measurement%
200	1.2	2.0
1000	1.1	2.0
